# *Pneumocystis jirovecii* Pneumonia in Critical Care: Evolving Host Populations, Diagnostic Uncertainty, and Management Controversies

**DOI:** 10.3390/microorganisms14102372

**Published:** 2026-10-16

**Authors:** Anna Naito, Joanna Kondratowicz, Sofia Hanger, Despoina Koulenti, Antonios Katsounas

**Affiliations:** 1 Adult Critical Care Directorate, St. George’s Hospital, St. George’s University Hospitals NHS Foundation Trust, London SW17 0QT, UK; anna.naito@nhs.net (A.N.); sofia.hanger@stgeorges.nhs.uk (S.H.); 2 Department of Renal Medicine, Heartlands Hospital, University Hospitals Birmingham NHS Foundation Trust, Birmingham B9 5SS, UK; j.kondratowicz@nhs.net; 3 Department of Critical Care Medicine, King’s College Hospital NHS Foundation Trust, London SE5 9RS, UK; 4 Frazer Institute, The University of Queensland, Brisbane, QLD 4102, Australia; 5 Faculty of Medicine, University of Thessaly, 41500 Larissa, Greece; 6 School of Health Science, University of Ioannina, 45110 Ioannina, Greece; 7 Department of Medicine, Knappschaft Kliniken University Hospital Bochum, Ruhr University Bochum, 44892 Bochum, Germany; antonios.katsounas@rub.de

**Keywords:** *Pneumocystis jirovecii* pneumonia, critical care, immunocompromised host, non-HIV, diagnosis, trimethoprim-sulfamethoxazole

## Abstract

*Pneumocystis jirovecii pneumonia* (PCP) remains a major cause of acute respiratory failure in immunocompromised patients and is increasingly encountered in critical care outside the traditional human immunodeficiency virus (HIV) population. This narrative review examines the evolving epidemiology, diagnosis, management, prophylaxis, and prognosis of PCP in critically ill adults. The epidemiological landscape has shifted toward patients with haematological and solid malignancies, solid organ or haematopoietic stem-cell transplantation, autoimmune and inflammatory diseases, and exposure to corticosteroids or other immunosuppressive therapies. Emerging data also implicate states of immune dysregulation such as cirrhosis. Diagnosis remains challenging because *Pneumocystis* cannot be routinely cultured and molecular detection alone cannot reliably distinguish colonisation from active infection. Accordingly, respiratory polymerase chain reaction (PCR) results require interpretation alongside host factors, imaging, serum β-D-glucan, microscopy, and, increasingly, metagenomic sequencing. Trimethoprim-sulfamethoxazole remains first-line therapy. Lower-dose regimens may reduce toxicity while maintaining efficacy, but current evidence is largely retrospective and prospective trials are needed. The benefit of adjunctive corticosteroids in non-HIV PCP remains uncertain, with conflicting observational data and no definitive mortality benefit demonstrated in the first randomised trial. Echinocandins remain investigational adjuncts, whereas venovenous extracorporeal membrane oxygenation may provide rescue support in selected patients with refractory acute respiratory distress syndrome. Prophylaxis is effective but remains inconsistently applied in several at-risk groups. Mortality among intensive care unit (ICU) patients remains high, particularly in non-HIV PCP, and varies substantially according to underlying disease, immune phenotype, co-infection, and severity of respiratory failure. Improved risk stratification and adequately powered prospective studies are priorities.

## 1. Introduction

*Pneumocystis* organisms were first reported in 1909 by the Brazilian physician Carlos Chagas. He originally suspected it was a protozoan and a form of *Trypanosoma cruzi*, the other organism he discovered [1]. It was not until 1988 that it was reclassified as a fungus [2]. The organism was originally named *Pneumocystis carinii*. However, as it was recognised that *Pneumocystis carinii* was not a single species but a group infecting various hosts, the species infecting humans was named *Pneumocystis jirovecii* in 1999 [3]. *Pneumocystis carinii* refers to the species infecting rats. Despite this reclassification, the abbreviation PCP (*Pneumocystis* pneumonia) remains widely used for human *Pneumocystis jirovecii* pneumonia [1]; for consistency with the contemporary critical-care literature, PCP will be used throughout this review.

PCP is an airborne-transmitted disease and tends to affect immunocompromised patients. Before the human immunodeficiency virus (HIV) and acquired immunodeficiency syndrome (AIDS) epidemic of the 1980s, the disease was rarely seen, with most early reports from the 1950s post-war period describing cases in malnourished children [4]. In 1981, the United States (U.S.) Center for Disease Control (CDC) published an article in its Morbidity and Mortality Weekly Report (MMWR): *Pneumocystis* Pneumonia—Los Angeles. It described five cases of PCP in five young, gay men [5]. This article marked PCP as the first AIDS-defining opportunistic infection recognised by the CDC.

The emergence of effective antiretroviral therapy (ART) and *Pneumocystis* prophylaxis in patients with HIV has led to a reduction in PCP rates among these individuals. However, there has been a rise in cases among other immunocompromised patients, including those with malignancies and autoimmune diseases, as well as those treated with immunosuppressive medications and glucocorticoids [6,7].

Despite improvements in the treatment of PCP, the mortality rate remains high, especially in non-HIV cases (ranging from 30% to 60%) and patients often warrant admission to intensive care units (ICUs) [8]. As of 2024, approximately 30% to 40% of those who develop PCP are HIV-positive [9]. HIV-infected patients with PCP were slightly less frequently admitted to the ICU (18.1%) compared to those without HIV (20.7%) between 2014 and 2019 [10]. Given the increasing heterogeneity and ongoing high mortality of critically ill patients with this disease, it is essential to review the literature on the epidemiology, diagnostics, management, prophylaxis, and prognostic factors of PCP patients in the critical care context [11].

Host immune phenotype also matters beyond the HIV/non-HIV distinction. In an unsupervised analysis of 481 patients with PCP, Gaborit et al. identified three clinical and biological clusters: an HIV-associated cluster with the most favourable survival; a cluster comprising haematological malignancies, immune-mediated inflammatory diseases and solid tumours, with greater inflammation, organ dysfunction and the poorest survival; and a predominantly solid-organ-transplant cluster [12]. These findings support assessing underlying disease, immunosuppressive exposure and inflammatory response together. Because all participants had PCP, this analysis describes phenotypes and prognosis after infection, rather than the relative risk of acquiring PCP.

### Scope and Literature Selection

This narrative review provides a clinically focused synthesis of PCP in adult critical care. The literature informing the review was supplemented by targeted searches of PubMed-indexed literature and primary journal and guideline websites, last updated on 9 October 2026. Search terms combined *Pneumocystis jirovecii* or *Pneumocystis carinii* pneumonia with critical care, immune phenotype, diagnosis, treatment and prophylaxis, including searches addressing specific host populations. No lower publication-date limit was imposed. Selection prioritised relevance, study design, and diagnostic definitions, with recent evidence considered alongside foundational reports. Case reports were used only to illustrate uncommon presentations. The literature selection was not systematic, and no formal risk-of-bias assessment was undertaken.

## 2. The Changing Epidemiology of PCP

Although PCP is historically known as an AIDS-defining opportunistic infection, it now affects an increasing spectrum of immunocompromised patients. These patients may have dysregulated immune systems due to the illness itself, or immunosuppressive therapy used to treat the underlying condition. There is also increasing evidence that PCP can affect patients who are deemed immunocompetent. This section details how the landscape of PCP epidemiology has changed over the last twenty years.

### 2.1. PCP in HIV Immunocompromised Populations

PCP still affects patients with HIV and typically presents when CD4 counts fall below 200 cells/μL. Prior to the advent of ART and PCP prophylaxis, 70–80% of those with AIDS historically developed PCP [13]. Although this has substantially reduced to <1 case per 100 person-years with HIV treatment in developed countries, mortality remains significant at 10–20% [14].

The epidemiology of PCP in developing low- and middle-income countries is less well described. A systematic review on PCP prevalence in Africa showed a 22% PCP prevalence by polymerase chain reaction (PCR) and 15% by microscopy in symptomatic individuals [15]. These rates are significantly higher than the 3.5% prevalence quoted in the U.S. [16]. Due to poor-quality studies, limited resources for testing, and the presence of concomitant opportunistic infections, prevalence rates in developing countries may be underestimated.

PCP remains a significant illness in HIV patients, with 17.8% to 22.6% of PCP cases in high-income countries occurring in HIV-infected individuals [17,18] and an increasing number of cases requiring ICU admission [10].

### 2.2. PCP in Non-HIV Immunocompromised Populations

In the last two decades, there has been an increase in PCP cases among patients without HIV, with a higher mortality rate compared to those with HIV (30–40% vs. 10–20%, respectively) [19]. This shift has led to a greater focus on understanding the overall immunosuppressive state of patients, which may be influenced by both the underlying disease and the medications used to treat it [20].

#### 2.2.1. Haematological or Solid Malignancy and Transplantation

The main non-HIV, non-iatrogenic risk factors for PCP include haematological malignancies, solid organ or haematopoietic stem-cell transplants, solid malignancies (most commonly lung and breast cancers), and autoimmune and inflammatory diseases [21,22].

Haematological malignancies, particularly lymphomas, confer a much higher risk of PCP compared to solid tumours [8,22].

PCP in solid organ transplant (SOT) patients has been most extensively studied in renal and liver transplant recipients. The epidemiology has shifted from early-onset (2–4 months post-transplant) to late-onset PCP (>12 months post-transplant), likely due to the 6–12 months of PCP prophylaxis typically administered after transplant [23]. Late-onset PCP has also been associated with acute T-cell-mediated rejections and co-infections with viruses such as severe acute respiratory syndrome coronavirus 2 (SARS-CoV-2) or cytomegalovirus (CMV) [24,25]. Post-transplant lymphoproliferative disorder (PTLD) was associated with increased risk of late-onset PCP. In this group, lymphopenia in the preceding 6 months, CMV infection, and previous rituximab use were also associated with increased risk of PCP [23].

A US study of 279 orthotopic heart transplant patients from 2008 to 2017, with a median follow-up of 3 years, found no cases of PCP; the researchers attributed this to the use of PCP prophylaxis for 12 months post-transplant and appropriate infection control protocols, although this does not explain the lack of late-onset PCP cases [26].

Delayed PCP after the early post-transplant period is also seen in allogeneic haematopoietic stem-cell transplant (HSCT) patients, where PCP occurred at a prevalence of 1% and a median of 11.5 months post-transplant [27].

#### 2.2.2. Autoimmune and Inflammatory Disease

In patients with autoimmune conditions, PCP has been reported in those with granulomatosis with polyangiitis (GPA), polyarteritis nodosa, polymyositis or dermatomyositis, systemic lupus erythematosus (SLE), and rheumatoid arthritis (RA), especially those that have pulmonary complications such as interstitial lung disease [19,28]. Studies specifically evaluating PCP risk for each autoimmune disease are limited [29]. In polymyositis or dermatomyositis, PCP is uncommon, but mortality among patients who develop PCP remains high at 33–60%, highlighting the importance of considering PCP early in the differential diagnosis [30].

Risk is not uniform across autoimmune diseases. Jiang et al. identified only five PCP cases among 6017 patients with SLE starting mycophenolate mofetil and/or cyclophosphamide, corresponding to 1.8 cases per 1000 person-years during the first six months of treatment; most affected patients had structural lung disease [31]. Prophylaxis was associated with adverse events, and these observational findings do not support universal prophylaxis solely on the basis of SLE. Decisions should instead account for lung disease, corticosteroid exposure and the overall intensity of immunosuppression.

High case fatality in selected autoimmune cohorts should not be interpreted as evidence that autoimmune disease itself independently worsens prognosis. In the French nationwide cohort of 4055 ICU patients reported by Kamel and Boulain, autoimmune inflammatory disease was not retained as an independent predictor of hospital mortality [10]. Differences from smaller cohorts may reflect case mix, concomitant lung disease, treatment exposure, illness severity and adjustment strategy; disease-specific susceptibility and prognosis after established PCP are separate questions.

Inflammatory conditions such as vasculitis also increase the risk of PCP, although they account for only 2.1% of all PCP patients [17]. Retrospective cohort studies identified antineutrophil cytoplasmic antibody (ANCA)-vasculitis as a risk factor [22]. A case series reported 7 patients with vasculitis (six ANCA-positive and one with giant cell arteritis) who developed PCP; all were on significant steroid therapy (>20 mg/day for at least 12 weeks) and none were receiving PCP prophylaxis [32].

Patients with inflammatory bowel disease (IBD), including ulcerative colitis (UC) and Crohn’s disease, also have an increased risk of PCP (10.6 per 100,000 person-years), which rises further in those receiving immunosuppressive therapy (32 per 100,000 person-years) [33]. A case–control study of UC patients in Japan identified age, use of three concurrent immunosuppressive therapies, and reduced lymphocyte counts as factors that additionally increased risk of PCP [34].

#### 2.2.3. Iatrogenic Immunosuppression

Many patients with the above conditions are treated with immunosuppressive or immunomodulatory therapy, which influences PCP risk.

Steroid therapy of >20 mg/day for >4 weeks increases risk of PCP [29]. It is often difficult to delineate whether the underlying disease itself or the immunosuppression contributes more to PCP risk; it is likely a combination of both. Certain conditions that require high-dose steroid therapy, such as giant cell arteritis, sarcoidosis, or minimal-change disease, do not appear to increase susceptibility [28,35], suggesting that disease-specific risk factors and/or concomitant immunosuppressive therapy may be more important determinants of risk.

Many immunosuppressive therapies significantly increase PCP risk when used in conjunction with steroids, including rituximab (monoclonal antibody against CD20 B-cells), methotrexate, thiopurines, tumour necrosis factor-alpha (TNF-α) inhibitors, immune checkpoint inhibitors, kinase inhibitors (e.g., ibrutinib), nucleoside analogues, and alkylating agents [36]. Interestingly, sulfasalazine may have a protective role against developing PCP [37].

Chimeric antigen receptor (CAR) T-cell therapy is a specialised immunotherapy that modifies a patient’s own T-cells to target specific cancer cells; it is now used in various haematological malignancies, namely B-acute lymphoblastic leukaemia (ALL), B-cell lymphomas, and multiple myeloma [38]. Up to 12% of patients receiving CAR T-cell therapy develop invasive fungal disease [39]. In a large-scale database analysis, PCP accounted for 11% of those cases [40]. These infections tended to occur late, >90 days after the CAR T-cell infusion. Studies postulate that B-cell aplasia, hypogammaglobulinaemia, and protracted CD4+ T-cell recovery, along with cessation of PCP prophylaxis around this time, may contribute to the risk of late-onset PCP [41,42].

### 2.3. PCP in Patients with Immune Dysregulation Without Classical Immunosuppression

#### 2.3.1. Lung

There is increasing evidence that patients who are presumed immunocompetent are also at risk of developing PCP. A Korean study explored 187 patients with PCP and identified a small subset (7.5%) who were deemed immunocompetent. Notably, 29% of these patients had chronic lung disease, while 50% had ischaemic heart disease. They tended to be older than their HIV-positive and non-HIV immunocompromised counterparts, experienced a higher 90-day mortality (71%), and required intubation more frequently [43]. Indeed, higher colonisation rates have been reported in interstitial lung disease (ILD) patients who were not on immunosuppressive therapy, reaching up to 34% [44]. Gálvez et al. did not find a relationship between various health conditions and PCP colonisation rates; however, overall PCP colonisation rates in their cohort were low [45]. Further research is required to determine how colonisation translates into the risk of active infection and which additional factors may contribute to immune dysregulation.

#### 2.3.2. Liver

Adding to this, a German study by Peschel et al. explored PCP cases in an ICU setting, focusing on those with liver cirrhosis; this study found that cirrhotic patients are at increased risk of both PCP and mortality, likely due to compromised immune function in cirrhosis, including reduced CD4+ T-cell and natural killer (NK) cell function [46].

Further evidence from Pulsipher et al. strengthens the understanding of the association between decompensated cirrhosis and PCP. Their study of 727 patients with proven or probable PCP revealed that among those with cirrhosis, 48% died, with a staggering 90-day mortality rate of ~58%. Mortality risk was significantly higher for patients with cirrhosis compared to those without, with an adjusted odds ratio (OR) of 4.08. Notably, cirrhotic patients were more likely to require ICU admission (87% vs. 42%) and renal replacement therapy (54.6% vs. 7.5%). These findings suggest that cirrhosis itself, even without additional immunocompromising conditions, is a significant and often underrecognised risk factor for PCP [47,48].

Additionally, in a separate cohort study, Pulsipher et al. found that the presence of cirrhosis was associated with a six-fold increase in the adjusted odds of mortality from PCP compared to other immunocompromised groups, including those with haematological malignancies and solid tumour cancers [49]. This finding underscores the heightened vulnerability of cirrhotic patients and calls for reconsideration of current PCP prophylaxis guidelines in these populations, where the incidence and severity of infection appear notably elevated.

Lafont Rapnouil et al. provide complementary evidence in severe alcohol-related hepatitis: across six ICUs, PCP was adjudicated in 11 of 103 patients (11%) admitted with hypoxaemic acute respiratory failure, comprising four proven and seven probable cases [50]. Nine additional patients had positive BAL PCR classified as colonisation. All 11 patients with PCP died in the ICU, but the small retrospective cohort, incomplete testing and competing causes of death preclude attributing mortality solely to PCP. Corticosteroid exposure was independently associated with BAL PCR positivity, an endpoint that included colonisation. These findings support a low threshold for PCP investigation in this presentation; they do not establish the incidence in all patients with alcohol-related hepatitis or the benefit of universal prophylaxis.

Taken together, these studies pose the question of whether a patient’s immune status and dysregulation have greater weight in PCP susceptibility than an underlying pathology.

### 2.4. PCP in Critically Ill Patients

Patients with underlying conditions that increase the risk of PCP have a similar likelihood of requiring ICU admission or intubation [51]. A multicentre multinational retrospective study identified 115 patients admitted to ICUs with proven or presumptive PCP, of whom 36% had HIV/AIDS, 31% had haematological malignancies, 24% had solid tumours, 15% had inflammatory diseases, and 5.2% had undergone solid organ transplantation. The study reported cumulative 30-day and 90-day mortality rates of 52% and 67%, respectively. Between 23% and 46% of PCP patients required mechanical ventilation [52,53,54]. Non-HIV patients with PCP experience longer hospital stays, higher rates of ICU admission and intubation, and increased mortality compared to patients with HIV (40–50% vs. 10–20%, respectively) [11,52].

Perret et al. analysed 249 PCP patients in a single centre in France, nearly half of whom had haematological malignancies; 37% were admitted to ICU [53]. Higher serum and bronchoalveolar lavage (BAL) CMV viral loads were associated with ICU admission. In India, the ICU admission rate for haematological and solid organ malignancy patients with PCP was higher at 43% [55]. Haematological patients may generally be at higher risk of severe illness, as seen in an allogeneic HSCT cohort, in which 50% required ICU admission with an overall mortality rate of 37% [27].

PCP has also been reported as an unusual cause of ventilator-associated pneumonia, including after elective cardiac surgery in a patient without traditional risk factors [56]. Such case-level evidence supports considering PCP in unexplained deterioration during mechanical ventilation, but does not establish its frequency as a cause of ventilator-associated pneumonia. A positive respiratory PCR still requires clinical and radiological corroboration, and late recognition does not by itself prove ICU acquisition.

Major limitations to the epidemiological studies include their retrospective design, inclusion of definite, probable, and possible PCP cases, and the lack of distinction between PCP colonisation and infection, as most rely on positive PCR from sputum, BAL, or serum, or on positive serum β-D-glucan (BDG).

## 3. Diagnosis of PCP

Diagnosing PCP is challenging for numerous reasons. The organism cannot be cultured or grown in vitro. The gold standard for definitive PCP diagnosis is direct visualisation under microscopy (historically using silver staining, and more recently immunofluorescence). Consequently, PCP is often a suspected diagnosis, based on a combination of clinical, radiological, and biochemical criteria. There are no universally accepted reference values for PCP due to variations between assay manufacturers and the difficulty of distinguishing colonisation from active infection.

### 3.1. Clinical and Radiological Presentation

Diagnostic criteria for PCP in non-HIV patients are currently based on the 2021 European Organisation for Research and Treatment of Cancer (EORTC) and Mycoses Study Group Education and Research Consortium (MSGERC) guidelines, which combine clinical, radiological, and laboratory factors (Table 1). These criteria were developed primarily to standardise research rather than to guide individual patient care [20]. Compared with HIV-associated PCP, non-HIV PCP typically has a more acute onset, more rapid progression, lower respiratory organism burden, and a more pronounced inflammatory response, frequently resulting in severe hypoxaemia. HIV-associated PCP generally has a more indolent presentation and higher organism burden, while cystic changes and pneumothorax are more commonly described in patients with HIV [20,57].

In patients with HIV, PCP typically presents with a subacute (1–2 week) onset of dyspnoea, non-productive (dry) cough, and fever; the absence of these symptoms has a high negative predictive value [14]. Similarly, the absence of exercise-induced oxygen desaturation increases sensitivity for ruling out PCP but has limited utility as it is non-specific [13]. In patients with HIV, McDonald et al. [13] suggest a diagnostic approach similar to the EORTC/MSGERC guidelines, with additional consideration of host factors such as ART status, CD4 count and percentage, and stage 3 HIV (AIDS) [14].

The typical radiographic (chest X-ray, CXR) pattern in these patients is diffuse, bilateral, symmetrical ground-glass interstitial infiltrates, often emanating from the hila in a butterfly pattern. Particularly in critical care, other diagnoses and syndromes exhibit similar radiological features, such as acute respiratory distress syndrome (ARDS), COVID-19 infection, and radiation pneumonitis; hence, they cannot be solely relied upon for diagnosing PCP [13].

In non-HIV patients, chest computed tomography (CT), particularly bilateral ground-glass opacities, and Pneumocystis PCR on BAL fluid are central components of diagnostic assessment. Neither establishes active PCP in isolation. Calvet et al. combined cohort-derived pre-test probabilities with published test-performance estimates in 2243 non-HIV immunocompromised patients with acute respiratory failure [58]. PCP frequency ranged from 2.0% without ground-glass opacities to 20.2% in patients with haematopoietic cell transplantation, ground-glass opacities and no prophylaxis. At the overall prevalence of 4.1%, modelled positive post-test probabilities were only 32.9% for PCR and 22.8% for BDG. These estimates illustrate how imaging and host context change test interpretation; they are not fixed predictive values transferable to every ICU or assay.

### 3.2. Conventional and Molecular Diagnostic Tests: Microscopy, PCR, BDG and Supportive Biomarkers

Biochemical tests include serum lactate dehydrogenase (LDH), serum BDG, and serum or respiratory PCR. Serum LDH alone has limited diagnostic value for PCP, with a pooled sensitivity of 67% (95% CI 59–75%) and specificity of 54% (95% CI 48–61%) in a mixed population of non-HIV and HIV patients [59]. In non-HIV patients, Sun et al. reported improved discrimination using a population-specific LDH threshold of 363 U/L, which differentiated probable/proven PCP from non-PCP with 80% sensitivity and 76% specificity [60]. However, LDH thresholds remain assay- and population-dependent and should not be used as standalone diagnostic criteria.

BDG is a component of the fungal cell wall, and serum assays have a much higher pooled sensitivity and specificity (91% and 79%, respectively) [61]; however, BDG has a high false-positive rate, as it can be raised in PCP colonisation, other fungal infections, in those treated with certain antimicrobials/intravenous immunoglobulins/albumin, and in patients undergoing renal replacement therapy [13]. Sensitivity is higher in those with HIV than those without HIV (94% vs. 86%) with similar specificity [20]. A negative BDG is likely to be useful in a context of low suspicion of PCP, where the post-test probability of disease is <5% if the pre-test probability is <20%, although a negative BDG result is less reliable in certain population groups, such as patients with solid or haematological malignancies [62].

Multiple studies have suggested the use of BDG cut-offs, but with no consensus due to the use of different assays and population groups. A meta-analysis of the Fungitell assay found a sensitivity and specificity of 84% and 76%, respectively, at an 80 pg/mL cut-off [63]. Li et al. [64] evaluated serum BDG cut-offs for diagnosing PCP in non-HIV immunocompromised patients admitted to ICU. Out of 400 non-HIV immunocompromised patients admitted to a single centre in China with suspected pneumonia, 42% were diagnosed with PCP. The manufacturers’ cut-off for positive BDG was 150 pg/mL, but they found that an optimal cut-off of 252 pg/mL provided an improved specificity (from 67% to 79%) whilst sensitivity was slightly decreased (from 94% to 87%). As the cut-offs are manufacturer-specific, these cut-off values cannot be standardised or compared.

PCR testing for PCP is highly sensitive, especially when performed on bronchoalveolar lavage (BAL) fluid or induced sputum, but its specificity is limited by the challenge of distinguishing colonisation from active infection [13]. Quantitative PCR (qPCR) improves specificity by allowing estimation of fungal burden; lower cycle threshold (Ct) values indicate higher DNA copy numbers, i.e., a higher organism burden. Again, Ct values are highly manufacturer- and assay-specific and hence cannot be compared between laboratories. In BAL fluid, qPCR sensitivity approaches 98–100% and specificity ranges from 80 to 95% depending on cut-off values and patient population [59,65,66,67,68]. Induced sputum yields similar sensitivity but slightly lower specificity [66,69]. Serum PCR (cell-free DNA) is less established but shows high specificity in proven cases, though sensitivity is variable and not yet standard for routine diagnosis [70]. Host factors impact PCR interpretation and cut-offs. HIV-positive patients typically have higher organism loads, so cut-offs for distinguishing infection from colonization are higher than in HIV-negative or SOT patients. A semi-quantitative PCR Ct cut-off of 36 allowed differentiation between colonisation and infection with an overall sensitivity of 71% and specificity of 77%; in SOT patients, the same cut-off yielded a sensitivity of 95% and specificity of 83%, respectively [71,72]. In line with these observations, in HIV-positive patients, a cut-off of 1.5 × 10^4^ copies/mL in BAL fluid achieves high sensitivity and specificity, while in HIV-negative patients, lower cut-offs (e.g., 3.4 × 10^3^ copies/mL) are needed due to lower fungal burden [65,67,73]. SOT recipients and other non-HIV immunocompromised patients may require even lower thresholds, and clinical context is essential [68,73].

CD4 count is relevant for risk stratification in HIV [74,75]. In non-HIV patients, lymphocyte subpopulations and immunoglobulin levels are not reliable diagnostic markers for PCP. While low absolute lymphocyte counts (<700/µL) and use of corticosteroids/immunosuppressants are risk factors for PCP, CD4 count alone is not predictive. The lymphocyte count cut-off for UC patients with PCP was 570/μL [34]. Decreased CD3/4/8 counts have been associated with PCP infection [75,76] as well as increased interleukin (IL)-2, IL-4, IL-10, and IL-13 mRNA expression [77], but they may be more useful as part of a diagnostic model (see below). Decreased CD3, CD4, and CD8 T-cells, combined with elevated LDH, may support diagnosis, but these are not specific and should also not be used in isolation [75].

### 3.3. Distinguishing Colonisation from Active Infection

Differentiating colonisation from active infection remains a major challenge that affects interpretation of many diagnostic tests. A recent multicentre study found that PCP colonisation in non-HIV patients with severe pneumonia requiring ICU admission was an independent risk factor for increased 28-day mortality, even after adjustment for co-detected microorganisms and clinical features [78]. Colonised patients had higher rates of immunosuppression, lower lymphocyte counts, and more frequent pulmonary viral co-infections, including CMV, Epstein–Barr virus (EBV), and human herpesvirus 6B/7 (HHV-6B/7) [78]. These findings raise the question of whether colonised critically ill patients warrant closer attention, though current guidelines [28] do not recommend treatment or prophylaxis specifically for colonisation, and further prospective studies are needed to determine whether intervention in this group improves outcomes.

### 3.4. Emerging Diagnostics and Diagnostic Models

Metagenomic next-generation sequencing (MNGS) is an emerging diagnostic tool, which analyses nucleic acid fragments and compares them to reference genomes, enabling simultaneous identification of multiple microorganisms in a sample. It is particularly useful when conventional tests have failed to identify a culprit organism. Meta-analyses report pooled sensitivity of 96–97% and specificity of 94–96% for detecting PCP, which is higher than BDG and LDH [79,80]. Although promising, the test is currently costly, time-consuming, and resource-intensive, with no standardised interpretation criteria [81]. A major limitation is its inability to differentiate colonisation from infection–the same caveat as when using BDG or PCR testing [82]. However, recent studies suggest that quantitative sequence read thresholds may help distinguish active PCP from colonization, analogous to qPCR Ct cut-offs [54,81,83]. Sun et al. (2025) demonstrated that a sequence read count <37 makes a PCP diagnosis unlikely (sensitivity 91%, specificity 87.8%), though other studies have proposed higher thresholds (e.g., 355 reads), reflecting variability across assays and populations [54,83]. As with BDG and PCR, clinical context remains essential for interpretation.

Due to the recent surge in artificial intelligence (AI) and data science, a multitude of papers have published predictive models for PCP diagnosis. Many of these are based on single-centre data, and translation to widespread clinical utility remains to be seen. A risk prediction model in low-resource settings showed a 60.6% risk of PCP if an HIV-positive patient scored all six points (CXR compatible with likely or probable PCP, low oxygen saturations, and absence of severe anaemia (haemoglobin, Hb > 9 g/dL)), which is just higher than a toss of a coin [84]. More recent predictive models using MNGS combined with lymphocyte count, BDG, and LDH levels have demonstrated high discrimination with an area under the curve (AUC) of 0.892 (95% CI 0.855–0.929), though external validation is needed [54]. A nomogram to predict mechanical ventilation requirement in patients with PCP has also been developed, incorporating age, oxygenation index, and LDH as independent variables, with an AUC of 0.829 (training cohort) and 0.818 (validation cohort) [85]. Additionally, a clinical risk score (the “PCP Score”) incorporating SOT or HIV with CD4 ≤200 cells/µL, LDH, radiological pattern, and PCP prophylaxis status achieved a c-statistic of 0.79 and may help estimate pre-test probability to guide testing and empirical treatment [86].

Non-invasive diagnostics are improving. Induced sputum PCR has high pooled sensitivity (99%) and specificity (96%), and plasma cell-free DNA PCR offers a promising non-invasive option with 100% sensitivity and 93.4% specificity in proven cases, though availability remains limited [59,70]. The increasing use of metagenomic testing, predictive modelling, and refined diagnostic algorithms will be an important area to pursue for accurate and timely diagnosis.

### 3.5. A Practical Diagnostic Approach in the Critically Ill Patient

**Figure 1:**
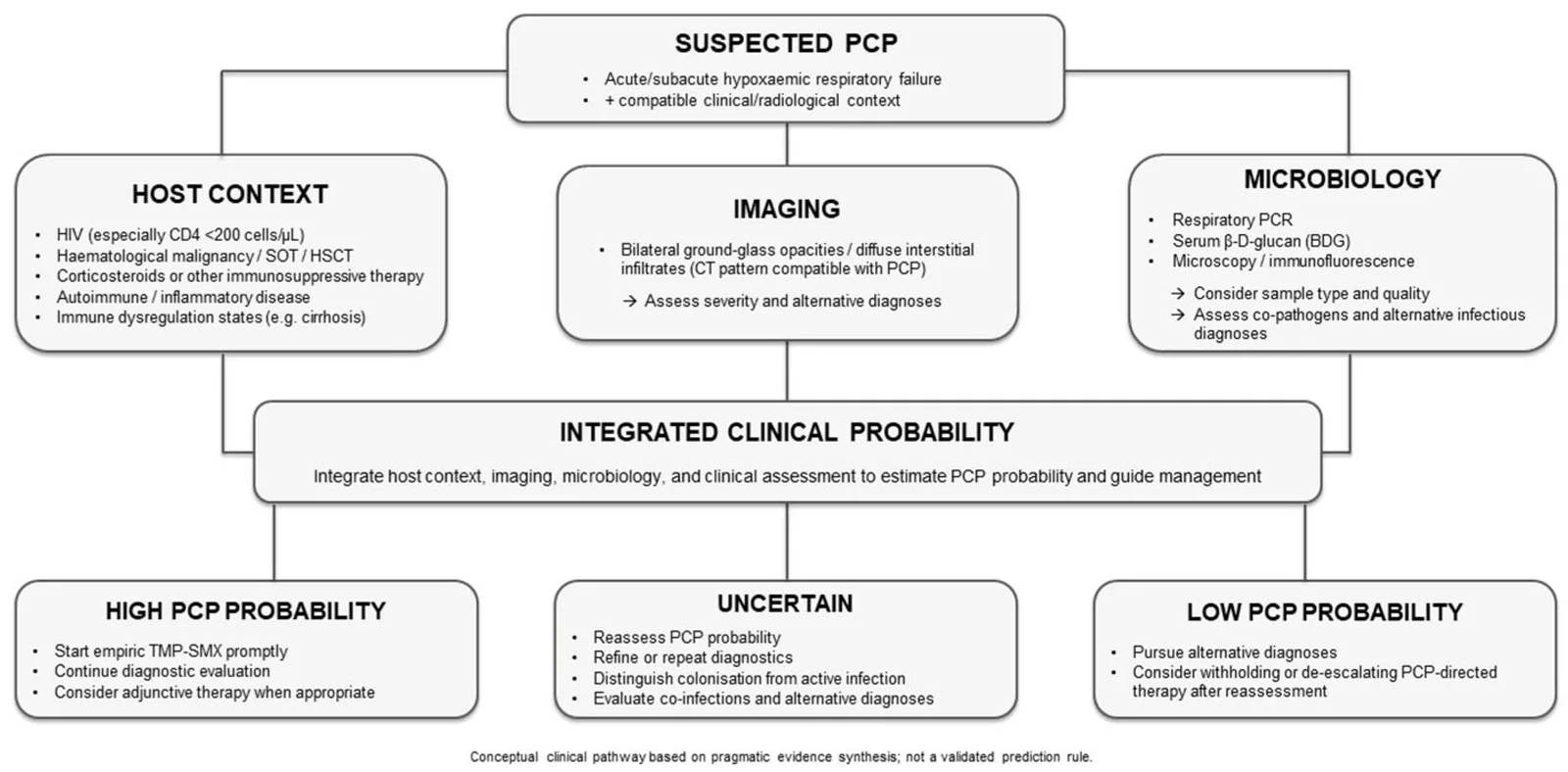
Clinical decision pathway for suspected *Pneumocystis jirovecii* pneumonia (PCP) in critically ill adults.

Combining serum BDG and respiratory PCR testing increases positive predictive value for PCP in critically ill patients. Concordant negative BDG and high-quality respiratory PCR results substantially reduce the probability of PCP, particularly when the pre-test probability is low or intermediate, but do not invariably exclude disease when clinical suspicion remains high [63,87,88]. A pragmatic clinical approach integrating host factors, imaging, microbiological findings, and clinical assessment to support PCP probability assessment and management decisions is summarised in Figure 1. This approach is particularly useful in oncology and ICU populations, where colonisation rates are high and single-test interpretation is insufficient [63,68,88]. One may argue that specimen access is less of an issue for intubated patients in critical care, but for non-intubated patients, the need for bronchoscopy remains a barrier.

The randomised diagnostic-strategy trial by Azoulay et al. provides context for selecting BAL rather than using it routinely in every patient [89]. Among 219 cancer patients with acute respiratory failure, early BAL plus non-invasive tests did not significantly increase intubation compared with non-invasive tests alone (35.4% versus 38.7%); the non-invasive strategy was not inferior for identifying the overall cause of respiratory failure. These overall results do not demonstrate equivalence specifically for excluding PCP. For suspected PCP, the potential diagnostic value of BAL PCR and microscopy must therefore be balanced against procedural risk, especially when non-invasive results are negative or inconclusive despite persistent clinical suspicion. Empirical treatment should not be delayed while arranging sampling in a patient with high clinical probability.

Conceptual framework integrating host factors, imaging, microbiology, and clinical assessment to guide PCP probability assessment and management decisions. Diagnostic interpretation requires consideration of colonisation, co-infections, and alternative diagnoses. This pathway is based on pragmatic evidence synthesis and is not a validated prediction rule.

## 4. Treatment of PCP

The optimal treatment strategy for PCP continues to be a topic of debate. Much of the randomised evidence supporting PCP treatment is extrapolated from trials conducted almost 30 years ago, predominantly in patients with HIV. Trimethoprim-sulfamethoxazole (TMP-SMX) remains the recommended first-line treatment, although alternative therapies may offer similar efficacy in selected patients. Additionally, low-dose TMP-SMX (TMP < 12.5 mg/kg/day) is emerging as a potentially less toxic approach in non-HIV patients. In a multicentre retrospective cohort, low-dose treatment was associated with similar survival and fewer adverse events compared with conventional dosing, although prospective randomised evidence is still lacking [90].

### 4.1. Timing of Treatment

The importance of timely treatment is highlighted in the multicentre prospective observational study by Kamel et al. in France [91]. This two-year study involved 49 adult ICUs across France, with 158 patients with PCP prospectively enrolled from 36 participating ICUs. Approximately 23% of the overall cohort had an autoimmune or inflammatory disease. Treatment initiation was delayed particularly among patients classified as having “other” immunocompromising conditions, most of whom had autoimmune or inflammatory diseases, compared with patients with solid or haematological malignancies. Among patients whose treatment was initiated in the ICU, starting therapy more than 96 h after ICU admission was associated with accelerated time to death in a propensity score-weighted time-to-event analysis (time ratio 6.75%, 95% CI 1.48–30.82; *p* = 0.014), supporting initiation of treatment as soon as PCP is clinically suspected [91].

### 4.2. TMP-SMX: Standard-Dose Versus Low-Dose Treatment

Despite the development of new drugs, TMP-SMX has remained first-line treatment for PCP. Much of the supporting randomised evidence is largely based on studies from the 1980–1990s, predominantly in men under 40 years of age with HIV. A recent systematic review and network meta-analysis of 14 randomised controlled trials involving 1788 participants found that, in direct comparison, no alternative treatment regimen showed significant superiority over TMP-SMX. In the network meta-analysis, clindamycin/primaquine, intravenous pentamidine and TMP-SMX ranked highest for prevention of treatment failure, whereas dapsone-TMP and intravenous pentamidine ranked highest for mortality reduction. Several alternative regimens also showed better tolerability than TMP-SMX, with significant reductions in treatment changes due to toxicity observed for dapsone-TMP, inhaled pentamidine, and atovaquone. The authors therefore concluded that TMP-SMX should be reassessed as the standalone first-line therapy for PCP in patients with HIV [92]. TMP-SMX is known to have significant toxicity, particularly at higher doses, prompting increasing interest in lower-dose treatment strategies.

Against this background, increasing attention has focused on whether lower-dose TMP-SMX can preserve efficacy while reducing treatment-related toxicity. Multiple retrospective cohort studies have examined this strategy. Huang et al. conducted a meta-analysis of studies comparing high- and low-dose TMP-SMX, with low-dose defined as less than TMP 15 mg/kg/day and high-dose referring to TMP 15–20 mg/kg/day [93]. Seven studies were analysed and low-dose TMP-SMX was associated with a significantly reduced mortality—12.9% compared with 25.9% in the high-dose group (OR = 0.49, *p* = 0.004). It was also found that patients who received a lower dose experienced fewer side effects and were more likely to complete the initial regimen, with fewer requiring second-line treatment. In a pharmacokinetic cohort, 32% of patients receiving a lower-dose TMP-SMX regimen versus 22% receiving higher-dose therapy achieved the prespecified peak sulfamethoxazole target; however, this difference was not statistically significant, and overall target attainment was poor [94].

A similar systematic review and meta-analysis in 2020 [95] appraised the effect of a low-dose TMP-SMX regimen, with three retrospective studies included. There was no significant difference in mortality between the high- and low-dose groups (absolute risk difference, −9%; 95% confidence interval [CI], −27% to 8%).

A multicentre retrospective cohort study [96] in patients with haematological malignancies found that low-dose TMP-SMX (7.5–15 mg/kg/day) was effective and safe for treating mild-to-moderate PCP, with no significant difference in 30-day mortality compared to standard dosing (14% vs. 15%) and no statistically significant difference in grade ≥ 2 adverse events (25% vs. 33%; *p* = 0.4). Similarly, Nagai et al. found no significant difference in 30-day mortality between low-dose (<12.5 mg/kg/day) and conventional-dose TMP-SMX in non-HIV PCP patients (6.7% vs. 18.4%; *p* = 0.080), but significantly fewer grade ≥ 3 adverse events in the low-dose group (29.8% vs. 59.0%; *p* = 0.005) [90]. A retrospective cohort study by Taniguchi had similar findings, comparing in-hospital mortality between low- and high-dose TMP-SMX groups in immunocompromised patients treated for PCP without HIV [97]. They found no significant difference in mortality, but the low-dose group had significantly increased treatment completion rates and reduced need for alternative agents.

Although lower-dose TMP-SMX has been associated with fewer adverse effects and, in some studies, lower mortality, whether improved tolerability mediates the observed survival association remains uncertain. The available evidence is predominantly retrospective and susceptible to confounding. The trial NCT04851015, currently in progress, will help to answer these questions by comparing TMP-SMX 10 mg/kg/day to 15 mg/kg/day with the composite primary outcome of change of treatment, new mechanical ventilation or death by day 21. Selected comparisons of conventional and reduced-dose TMP-SMX regimens are summarised in Table 2.

**Table 2:** Selected comparisons of conventional and reduced-dose TMP-SMX treatment for PCP.

Evidence and Population	TMP Dose in mg/kg/day	Outcomes and Interpretation
Conventional regimen [14]	15–20; SMX 75–100	Guideline-supported treatment dose. Monitor renal function, potassium and blood counts; adjust dosing for renal function.
Nagai et al. [90] Non-HIV cohort	<12.5 versus 12.5–20	30-day mortality: 6.7% versus 18.4% (*p* = 0.080); grade ≥ 3 adverse events: 29.8% versus 59.0% (*p* = 0.005). Observational comparison; non-significance does not establish equivalence.
Hammarström et al. [96] Haematological malignancies	7.5–15 versus >15–20	30-day mortality: 14% versus 15%; grade ≥ 2 adverse events: 25% versus 33% (*p* = 0.4). Evidence is most applicable to mild-to-moderate PCP; severe ICU disease remains uncertain.
Huang et al. [93] Seven-study meta-analysis	<15 versus 15–20	Mortality: 12.9% versus 25.9% (OR 0.49; *p* = 0.004); fewer adverse events at lower doses. Retrospective source studies, heterogeneous regimens and confounding limit causal interpretation.

Doses refer to the trimethoprim (TMP) component unless stated otherwise. Definitions of reduced dose differ between studies. Percentages are within-study comparisons and should not be compared directly across rows. SMX, sulfamethoxazole; OR, odds ratio. These data do not establish reduced-dose non-inferiority in severe PCP.

### 4.3. Adjunctive Echinocandins

Echinocandins have been used as adjunctive treatment for PCP; however, the evidence for their use is predominantly observational and heterogeneous, with no demonstrated mortality benefit in the non-HIV population. There is some emerging evidence for their use in select situations, although further prospective trials will be required to determine if they are a viable treatment option. A recent meta-analysis of seven studies [98] (1963 patients) found that TMP-SMX plus echinocandins significantly reduced mortality in patients with HIV (OR 0.44; 95% CI 0.28–0.70; *p* = 0.0005) but not in non-HIV patients (OR 1.00; 95% CI 0.57–1.76; *p* = 0.99). Treatment response rates were higher with combination therapy in both populations. Echinocandins may also serve as an alternative for patients intolerant to TMP-SMX, with case series showing clinical cure in the majority of patients treated with echinocandin plus clindamycin [99,100]. There are ongoing trials into the use of rezafungin as prophylaxis and as an option for treatment in combination with TMP-SMX [101].

### 4.4. Adjunctive Corticosteroids

The study by Kamel et al. in France [91] examined practice regarding steroid use and found that 64.4% of the cohort received steroids (including 82.8% of HIV patients). The mean daily dose was 80 mg of a prednisolone equivalent. While 6-month mortality did not differ between the groups receiving steroids and those who did not, a sub-analysis showed a significantly faster occurrence of death in patients without HIV who received steroids, though this observational finding is subject to confounding by indication.

The ongoing uncertainty regarding the role of steroids in patients with PCP who do not have HIV has been reviewed in several retrospective observational cohort studies. Many of these have significant limitations, including varying doses of steroid, the severity of illness within the patient population, and small sample sizes. Two retrospective cohort studies found a significant reduction in mortality with steroid use in this population [102,103]. The 2020 systematic review by Ding et al. included 16 retrospective observational studies and found that there was increased mortality in all non-HIV PCP patients (OR 1.37; 95% confidence interval 1.07–1.75; *p*  =  0.01). Further subgroup analysis revealed that non-HIV PCP patients with respiratory failure who were treated with corticosteroids had improved mortality (OR 0.63, 95% CI 0.41–0.95, *p* = 0.03). In addition, patients with hypoxaemia treated with low-dose corticosteroid (1 mg/kg/day prednisolone) had lower mortality (OR 0.61, 95% CI 0.39–0.94, *p* = 0.02) [104].

This was addressed in the first large randomised placebo-controlled trial in France [105]. This multicentre, double-blind trial randomised 226 HIV-negative immunocompromised patients with severe PCP to adjunctive intravenous methylprednisolone (30 mg twice daily on days 1–5, 30 mg once daily on days 6–10, and 20 mg once daily on days 11–21) or placebo: 218 patients were included in the intention-to-treat analysis. The trial did not demonstrate a statistically significant reduction in the primary outcome of 28-day mortality (21.5% with corticosteroids vs. 32.4% with placebo; 10.9-percentage-point absolute difference; 95% CI −0.9 to 22.5; *p* = 0.069). Among the secondary outcomes, 90-day mortality was significantly lower in the corticosteroid group, and the need for invasive mechanical ventilation was reduced among patients who were not intubated at randomisation. Adverse events, including secondary infections, were not increased with corticosteroid treatment.

Despite these findings, more recent observational studies have shown conflicting results. A propensity-matched multicentre study [106] found no significant reduction in 90-day mortality with early adjunctive corticosteroids in 350 HIV-negative patients, and corticosteroid recipients had longer mechanical ventilation duration. Similarly, a dose–response analysis [107] found that higher cumulative corticosteroid exposure was associated with increased 90-day mortality (hazard ratio, HR 1.01 per 100 mg prednisone-equivalent; 95% CI 1.00–1.02; *p* = 0.006) without improved respiratory outcomes. These findings suggest that doses exceeding trial-tested regimens should be approached with caution, and further work to identify which patients may benefit from adjunctive corticosteroids is required.

The evidence continues to demonstrate the uncertainty in the benefit of corticosteroids in this cohort and the importance of patient selection. Individual patient characteristics such as disease severity, inflammatory phenotype, concomitant conditions and the risks of further immunosuppression must all be considered when deciding the optimal treatment for the individual patient.

### 4.5. Extracorporeal Organ Support/VV-ECMO

With the high mortality rate secondary to PCP infection, it is unsurprising that clinicians continue to strive for effective treatments. Venovenous extracorporeal membrane oxygenation (VV-ECMO) can be an effective rescue therapy for immunocompromised patients with severe ARDS secondary to PCP. A 10-year multicentre study across four ECMO centres found that 80% of PCP patients (4 of 5) survived to hospital discharge, though ECMO duration was prolonged (median 30 days; interquartile range, IQR 21–43) compared to other fungal pneumonias [108]. A retrospective cohort study in China demonstrated similar results in immunocompromised kidney transplant recipients with severe ARDS secondary to PCP, with 80% survival in the ECMO group compared with 35% in the control group [109]. These studies had several potential confounding factors, including small sample sizes and single-centre designs; however, they point to the potential benefit of ECMO in this high-risk population when conventional therapies fail.

### 4.6. Immune Reconstitution Inflammatory Syndrome (IRIS)

IRIS related to PCP accounts for approximately 2.7–4% of IRIS cases following initiation of ART for HIV; however, it can be associated with significant morbidity. PCP-IRIS remains a diagnosis of exclusion and alternative causes for clinical deterioration, such as treatment failure, non-adherence and co-infections should be considered in the first instance. In a recent review of 51 published and previously unpublished cases, approximately 22% of patients with PCP-IRIS require admission to ICU for management of respiratory failure, highlighting the potential severity of this complication [110]. Risk factors for developing IRIS include a low CD4 count and initiation of ART within a short time of diagnosis of an opportunistic infection. Clinical manifestations and diagnostic tests are the same as for PCP, and IRIS should be suspected if there are persistent or recurrent fever, cough or shortness of breath. Evidence of the best approach to treatment of PCP-IRIS remains scant, with the latest recommendations stating that ART should be commenced within two weeks of PCP treatment [111]. The use of steroids specifically in PCP-IRIS remains unclear, although some advocate for high-dose steroids in those with high severity of illness or requiring ICU admission. The treatment duration of TMP-SMX remains 21 days, the same as for PCP [110].

In summary, the management of PCP, which has been largely unchanged for several decades, is currently under scrutiny. In the critically unwell patient with PCP, the evidence continues to support the importance of early diagnosis and prompt treatment initiation in improving mortality. TMP-SMX remains the first-line treatment while further prospective randomised trials examining the potential of a lower-dose regimen are undertaken. Adjunctive echinocandins are an additional area of research and may benefit patients with HIV but have not shown mortality benefit in non-HIV populations. For patients with severe refractory ARDS, VV-ECMO may be considered as a rescue therapy at experienced centres.

## 5. Prevention and Prophylaxis of PCP

With the advent of increasing numbers of patients receiving immunosuppressive treatment, there is a renewed focus on which patients benefit from PCP prophylaxis. This is an important consideration for ICU physicians, whose patients may be started on immunosuppression during their stay.

### 5.1. Established Indications

For people living with HIV, the NIH adult and adolescent opportunistic-infection guidelines recommend primary prophylaxis when the CD4 count is <200 cells/µL in those not receiving or starting ART [14]. For those already on ART, prophylaxis is indicated or restarted at CD4 <100 cells/µL regardless of viral load, or at CD4 100–200 cells/µL with detectable HIV RNA. TMP-SMX is preferred. Primary prophylaxis can be discontinued after a CD4 increase to ≥200 cells/µL for ≥3 months on ART; discontinuation may also be considered at CD4 100–200 cells/µL with sustained viral suppression for 3–6 months. A previous PCP episode is an indication for secondary prophylaxis until guideline-defined immune recovery.

For SOT recipients, the American Society of Transplantation Infectious Diseases Community of Practice recommends routine TMP-SMX prophylaxis for at least 6–12 months after transplantation [112]. Duration beyond this period should reflect the transplanted organ and ongoing immunosuppression, with prolonged or lifelong prophylaxis commonly used after lung transplantation. Treated rejection, lymphopenia and CMV infection should prompt reassessment of the need to extend or restart prophylaxis [8,112]. The end of routine prophylaxis does not eliminate the risk of late PCP.

Some bodies have clear guidelines, such as the ECIL guidelines [113], which mandate which haematology patients should receive prophylaxis (including ALL, allogeneic HSCT, treatment with alemtuzumab, fludarabine/cyclophosphamide/rituximab combinations, and >4 weeks of corticosteroid treatment). American Society of Clinical Oncology/Infectious Diseases Society of America (ASCO/IDSA) guidelines [114] recommend prophylaxis for patients receiving chemotherapy regimens associated with >3.5% risk for PCP, including those receiving ≥20 mg prednisone equivalents daily for ≥1 month.

### 5.2. The Prophylaxis Gap in Emerging Risk Populations

In other areas, such as rheumatology, there is ongoing research to establish which patients will benefit from PCP prophylaxis. Notably, a recent study found that 59% of non-HIV PCP cases should have been on prophylaxis according to existing guidelines, with the lowest compliance observed in haematological disease patients (72% without indicated prophylaxis), highlighting the gap between recommendations and clinical practice [115].

## 6. Outcomes and Prognostication of PCP

Prognosis in PCP infection remains poor in those admitted to the ICU. Most recent studies looking at non-HIV PCP show 30-day mortality rates of approximately 50–53% and 90-day mortality rates of up to 67% [11,116,117]. HIV-related PCP has a much-improved survival rate with current ART, and most studies reflect a better prognosis for this cohort compared to patients without HIV [10,118].

When discussing prognosis for PCP infection, it is important to recognise the heterogeneity of the groups of patients susceptible to this infection without HIV. Some studies focus on distinct subsets of immunosuppressed patients. Because the number of patients with these conditions who develop PCP is often small, studies can lack sufficient power to draw robust conclusions. Conversely, many papers do not stratify patients sufficiently (dividing them into HIV-positive or HIV-negative only), potentially leading to over-generalisations.

### 6.1. HIV Versus Non-HIV

The survival rate has been drastically improved, with a 5-year survival rate of 73% for patients diagnosed with HIV-PCP between 2000 and 2013, and survival likelihood of 88% among ART-adherent patients [118]. The exception to this is the increased risk of developing IRIS if commenced on combination ART early in infection. There is also evidence that patients with HIV who contract PCP have increased rates of restrictive lung pathology and bronchiectasis, as well as increased CMV infection rates due to treatment with corticosteroids.

Prognosis of HIV-positive patients with PCP in regard to mortality appears to be better than the prognosis of HIV-negative patients with PCP [91] though some studies have shown a non-significant difference after adjusting for confounders [11,17,18]. The poorer prognosis in non-HIV patients may in part be explicable by age, increased likelihood of corticosteroid use, underlying diseases, or frailty.

There are fewer recent studies looking specifically at prognostic factors of PCP patients with HIV in the current climate of ART use. It should also be noted that PCP infection is significantly more prevalent in patients with CD4 <50 cells/mm^3^ [119], and these patients are more susceptible to respiratory co-infections with different pathogens, owing to greater immunocompromise [120].

### 6.2. Host Factors and Underlying Disease

In non-HIV patient subsets, patients with pre-existing interstitial lung disease appear to have a significantly worse prognosis [121]. In one study, connective tissue disease (CTD)-ILD patients with PCP had a mortality rate of 49.1%, whereas those with CTD but no ILD had a mortality rate of 33.7% (*p* = 0.019) [122]. In another study, microscopic polyangiitis (MPA) was associated with increased mortality in patients with vasculitis-PCP, possibly due to a higher prevalence of ILD in this group [123]. It has also been shown that risk factors for mortality differ between those with and without chronic pulmonary disease, making interpretation of procalcitonin (PCT) levels, neutrophil counts and Hb challenging as reliable markers for prognostication [124].

There have been some studies looking at smaller subgroups of patients for whom risk factors for PCP are distinct in regard to corticosteroid use. For example, those with anti-melanoma differentiation-associated gene 5 antibody-positive dermatomyositis (MDA5-DM) are prone to opportunistic infections including PCP, and there is an association between rapidly progressive ILD and mortality, whereas steroid use is actually associated with lower levels of mortality [125]. These differences may also be diluting observed effects in non-stratified subsets of patients with non-HIV PCP.

Metastatic solid malignancies and chronic liver disease have shown an independent association with increased 30-day mortality (HR 3.49; 95% CI 1.71–7.13 and HR 2.44; 95% CI 1.03–5.80, respectively) [11]. In lung cancer patients, the use of immune checkpoint inhibitors offers survival benefits but appears to increase the risk of mortality from PCP [126].

A retrospective study of 506 patients with Hodgkin’s lymphoma, receiving chemotherapy in a single centre, had an incidence of PCP of 6.2% and mortality of 8% at 30 days after PCP [127].

A study of 57 patients with systemic lupus erythematosus (SLE) and PCP infection between 2014 and 2022 showed a mortality rate of 38.6% with a number of factors including higher LDH, C-reactive protein (CRP), diabetes mellitus/hyperglycaemia, and lower complement component 3 (C3) and Hb identified as risk factors [128].

In a case–control study of SOT recipients from 2000 to 2020 (most commonly renal transplant, which made up 55.2% of patients), where 67 PCP cases were matched to 134 controls, PCP was significantly associated with mortality within 90 days of diagnosis (*p* < 0.001) as well as 90-day death-censored renal allograft loss (*p* = 0.026). Among PCP patients, 44.8% required admission to an ICU and 24 (35.8%) required invasive mechanical ventilation [23].

The low number of patients in many of these studies makes it difficult to draw robust conclusions.

A multicentre retrospective observational study from January 2011 to April 2019 looked at respiratory BAL samples that tested positive for *Pneumocystis jirovecii* and met criteria for PCP rather than just colonisation. Of these, they identified 155 patients (47.3%) with a proven and 173 (52.7%) probable PCP. PCP was proven when *Pneumocystis jirovecii* asci (cysts) were found at direct microscopic examination. Probable PCP was determined by a negative direct mycological examination but a positive *Pneumocystis jirovecii* real-time qPCR in a respiratory sample, associated with a compatible clinico-radiological presentation. A higher 90-day mortality rate was independently observed in patients without HIV but with immune-mediated inflammatory diseases, solid tumours, immunosuppression with previous corticosteroid use (daily dose ≥ 10 mg) and the most favourable prognosis was observed in those with HIV. Interestingly, patients who were HIV-positive had a higher rate of proven vs. probable PCP diagnosis [22].

This disease-associated prognostic signal is not uniform across datasets: autoimmune inflammatory disease was not an independent hospital-mortality predictor in the nationwide ICU analysis by Kamel and Boulain [10]. The difference between a clinically characterised multicentre PCP cohort and an administrative cohort restricted to ICU admissions limits direct comparison of their effect estimates.

With regard to corticosteroid use, several studies have previously shown an association between previous steroid use in non-HIV patients and increased risk of death from PCP [19]. In a multicentre study of 37 patients with PCP infection and rheumatoid arthritis, higher prednisolone dose at the time of infection was associated with increased mortality [129]. Similarly, a study of 8021 HIV-negative patients with PCP showed significantly increased mortality in those who had received prior corticosteroid therapy at 10 weeks (16% versus 9%, *p* < 0.0001) and one year (23% versus 14%, *p*  <  0.0001) as well as higher BDG levels, perhaps indicating higher fungal burden [130]. More recently, a multicentre cohort study demonstrated that higher steroid dose in the 1–40 days before PCP onset was strongly associated with increased 90-day mortality in non-HIV PCP patients, independent of age, sex, and underlying disease [131]. It should be noted that these results do not necessarily imply causation as higher doses of corticosteroids may be related to more aggressive underlying disease which may confound results.

It is clear that PCP is still associated with very high mortality rates of 30–50% depending on patient subgroup. In order to better prognosticate for PCP, more data and larger patient subsets are warranted. Prognoses, especially in regard to steroid use, may need to be stratified according to underlying disease.

### 6.3. Co-Infection and Respiratory Microbiome

PCP co-infection with other pathogens, including viruses such as CMV (which is commonly seen in SOT recipients), is related to increased mortality rates in a number of studies [18,132]. Respiratory CMV co-infection at PCP diagnosis was independently associated with increased 90-day mortality (*p* = 0.02), specifically in HIV-negative patients [18]. CMV detection in BAL with viral load ≥ 3 log copies/mL was associated with significantly higher ICU admission rates (78.4% vs. 28.4%, *p* < 0.001) [53]. Indeed, CMV pneumonia is independently associated with a high mortality rate, with a study of patients in China with severe pneumonia showing similar 90-day mortality rates between CMV and PCP, with both being higher than for non-CMV pneumonias [133]. PCP patients co-infected with pulmonary aspergillosis also have a high mortality rate (49% in one study) [134].

This does not necessarily imply causation as it is likely that those who are more immunocompromised are also more prone to infection with multiple pathogens and it may be the immunocompromise rather than the co-infection that is linked to worse outcomes.

A limiting factor in many studies is that patients included in data analyses include those who are both ‘proven’ PCP and ‘probable’ PCP cases. It may be that a subset of these patients who do not have microbiological confirmation of PCP infection have other respiratory causes for symptoms, including other respiratory viruses. In addition, some patients classified as having confirmed PCP with co-infection may have deteriorated primarily because of another pathogen, while the detection of *Pneumocystis jirovecii* in BAL fluid may have reflected colonisation rather than active infection. Interestingly, there were no differences observed in mortality rates between proven and presumptive diagnosis in one study [11].

Metataxonomic sequencing of respiratory microbiota has altered the landscape of diagnostics in this field of research and has given increased weight to the suggestion that not only co-infection but colonisation profiles are associated with variability in prognosis. A recent 2025 study by Del Prete et al. looked at patients admitted to a centre in Italy between 2020 and 2021 with PCP. BAL samples were taken and showed that severe PCP was associated with lower bacterial diversity, an increase in *Prevotella* and a decrease in *Neisseria*, though the sample size was relatively small with 24 participants undergoing BAL [135]. Likewise, *Pneumocystis jirovecii* colonisation may be associated with increased 28-day mortality in patients with severe non-PCP [78].

### 6.4. Physiological and Laboratory Predictors

The variables that have consistently been shown to increase mortality risk in non-HIV PCP infection relate to CD4 count and BAL lymphocyte/neutrophil counts. A study of 178 non-HIV PCP patients over 2 years compared BAL fluid results in those who died versus those who survived following infection. It found a significantly higher neutrophil count and significantly lower lymphocyte count in the non-survivor group, suggesting this may be a predictor of mortality [136]. Lower CD8+ T-cell counts and recent steroid withdrawal (≤5 days from corticosteroid withdrawal to symptom onset) have also been associated with poor outcome [116]. A retrospective study of 46 patients between 2018 and 2020 also found that compared with survivors, non-survivors had a higher acute physiology and chronic health evaluation II (APACHE II) score, PCT/LDH levels, neutrophil-lymphocyte ratio and a lower arterial partial pressure of oxygen to fraction of inspired oxygen (PaO_2_/FiO_2_) ratio and CD4+ T-cell count. Co-infection with another organism was an independent risk factor of poor prognosis [116,137].

One study suggests that prognosis is worse in patients with a high CRP (>135 mg/L) and to a lesser degree a hypo-inflammatory state (CRP < 30 mg/L) compared to a normo-inflammatory state at 48 h of admission. There were no significant differences in white cell counts or steroid treatment between the groups [138].

As with other causes of respiratory failure, poor prognostic markers for PCP-infected individuals admitted to ICU include high arterial carbon dioxide partial pressure (PaCO_2_) and lower arterial pH within the first 24 h of ICU admission. Though less significant, a higher carboxyhaemoglobin (HbCO) level may also be indicative of poorer prognosis, perhaps because it reflects more severe lung inflammation [139].

### 6.5. Prognostic Scores and Their Limitations

The combination of confusion, urea, respiratory rate, blood pressure, age ≥ 65 (CURB-65 score), high-resolution computed tomography (HRCT) score, and white blood cell (WBC) count may correlate with mortality risk from PCP [140].

There are a number of large-scale studies, mainly in China, that attempt to create scores to predict mortality/severity of PCP, though many of them are not validated clinically [141] and are complex to calculate, making their utility in clinical practice uncertain. For example, Chung Yi Koh et al. designed a risk score comprising age (>65), sex (male sex associated with increased risk), and LDH (>770 U/L) to help with prognostication in HIV-negative PCP patients. It was shown to be better at negative predictive value for mortality than positive [142]. A team at Peking Union Medical College Hospital also designed a tool to evaluate risk of ICU admission and death comprising eight factors including shock, invasive mechanical ventilation, chronic lung disease, respiratory rate (RR), blood urea nitrogen (BUN), LDH, CMV infection, and age, displayed as a nomogram [143].

## 7. Conclusions and Future Priorities

PCP is an opportunistic fungal infection that can cause acute respiratory failure necessitating critical care admission. This review aimed to consolidate the shifting epidemiology and emerging evidence regarding diagnostics and management of PCP.

*Epidemiology:* The landscape of PCP epidemiology has changed significantly over the last twenty years. Previously felt to be affecting predominantly HIV patients as an AIDS-defining illness, PCP now occurs in those with varying immunocompromised states. With a rising prevalence of PCP affecting the non-HIV population, it is important to consider HIV and non-HIV-associated PCP as separate clinical entities.

*Diagnosis:* PCP diagnosis is difficult because *Pneumocystis* cannot be routinely cultured in vitro and colonisation cannot easily be distinguished from active infection. Although BDG and PCR are common biochemical investigations, these tests are assay- and manufacturer-specific with no standardisation between laboratories. Using a multimodal approach of clinical, radiological, and biochemical investigations remains the mainstay, and having a high index of suspicion is paramount.

*Treatment:* Despite significant clinical experience and recent observational studies, the optimal management of PCP remains incompletely defined, particularly outside the HIV population from which most historical evidence derives. TMP-SMX continues to be the first-line treatment.

*TMP-SMX:* Low-dose TMP-SMX represents one of the more promising developments in recent years, although all supporting evidence remains retrospective in nature. This strategy should therefore be regarded as promising rather than established practice, pending the results of ongoing prospective trials.

*Adjunctive echinocandins:* Adjunctive echinocandins may have a role in patients with HIV but lack demonstrated benefit in non-HIV PCP.

*Extracorporeal support:* VV-ECMO offers a viable rescue option in select patients with refractory ARDS, although evidence remains limited to small observational cohorts.

*Adjunctive corticosteroids:* The role of adjunctive corticosteroids in non-HIV PCP is similarly unresolved. Corticosteroid use should therefore be individualised, guided by disease severity and the risks of additional immunosuppression, rather than applied uniformly. There are specific subgroups, such as those with MDA5-DM, in whom corticosteroids may be associated with benefit. However, the majority of data on non-HIV patients suggests recent steroid use prior to PCP diagnosis is associated with increased mortality as is steroid withdrawal in the preceding days before symptom onset. This may be a consequence of immunosuppression or could reflect greater underlying disease severity warranting steroid use.

*ICU prognosis:* Prognosis remains poor for patients admitted to ICU with PCP infection with a 30-day mortality rate between 50% and 53%. It should be emphasised that PCP prognosis varies significantly according to underlying diagnosis and host factors within non-HIV subgroups.

Subgroups of patients without HIV are heterogenous and usually small, making firm conclusions about prognosis challenging. In non-HIV patients, it appears that concurrent ILD, vasculitis, metastatic solid cancers and receiving a SOT are risk factors for poorer outcomes. Various scoring systems have been created to predict prognosis in PCP patients without HIV but are not verified. Factors associated with poor outcome include lower CD4 cell count, higher neutrophil/lower lymphocyte count and co-infection with another organism. Higher PaCO_2_ and lower arterial pH in the first 24 h of critical care admission are also associated with poor prognosis.

*Research priorities:* Overall, PCP management is entering a period of re-evaluation. Further prospective, adequately powered trials are needed to clarify the optimal dosing of TMP-SMX and the role of adjunctive therapies, particularly corticosteroids, in the growing non-HIV immunocompromised population.

PCP remains a life-threatening fungal infection with heterogeneity in patient population, diagnostic challenges, lack of clear consensus in management options, and high mortality rates in those admitted to critical care. Clinicians must remain vigilant in suspecting PCP in patients requiring critical care with undifferentiated respiratory failure and ongoing studies on optimal management and prophylaxis are crucial to tackle the above issues.

## Figures and Tables

**Table 1:** PCP diagnosis for non-HIV patients as per EORTC/MSGERC 2021 guidelines (Adapted from [20]).

Proven PCP	Clinical and radiological criteria + demonstration of *Pneumocysitis jirovecii* by microscopy using conventional or immunofluorescence staining in tissue or respiratory specimens
Probable PCP	Appropriate host factors and clinical and radiological criteria plus either: - Amplification of Pneumocysitis jirovecii DNA by quantitative real-time PCR in a respiratory specimen - Detection of β-D-glucan (BDG) in serum (alternative method; another invasive fungal disease and a false-positive result should be ruled out)

## Data Availability

No new data were created or analyzed in this study. Data sharing is not applicable to this article.
